# Short-Term Exposure to MPs and DEHP Disrupted Gill Functions in Marine Bivalves

**DOI:** 10.3390/nano12224077

**Published:** 2022-11-19

**Authors:** Yanfei Zhou, Yanping Li, Wenlu Lan, Hao Jiang, Ke Pan

**Affiliations:** 1 Shenzhen Key Laboratory of Marine Microbiome Engineering, Institute for Advanced Study, Shenzhen University, Shenzhen 518060, China; 2 Key Laboratory of Optoelectronic Devices and Systems of Ministry of Education and Guangdong Province, College of Physics and Optoelectronic Engineering, Shenzhen University, Shenzhen 518060, China; 3 Marine Environmental Monitoring Center of Guangxi, Beihai 536000, China; 4 Key Laboratory of Aquatic Botany and Watershed Ecology, Wuhan Botanical Garden, Chinese Academy of Sciences, Wuhan 430074, China

**Keywords:** microplastics, organic pollutants, bivalves, oxidative damage, synergic effects

## Abstract

The synergistic impact of microplastics (MPs) and organic pollutants remains poorly understood in the marine environment. This study aimed to assess the toxicity of polypropylene microplastics (PS) and/or di-(2-ethylhexyl) phthalate (DEHP) on marine clams. Both *Ruditapes philippinarum* and *Tegillarca granosa* were exposed to PS and DEHP individually and combined at environmentally relevant concentrations for 48 h. The filtration rate, antioxidant enzymes activity, lipid peroxidation, reactive oxygen species accumulation, and histological alterations were evaluated. Our results show that single or co-exposure to MPs and DEHP significantly decreases the filtration rate in both type of clams, but the latter exhibited stronger inhibition effect. Close examination of accumulation of reactive oxygen species and related biomarkers revealed that combined exposure exerts greater oxidative stress in the cells, which causes more serious histopathological damage in the gills of the bivalves. Our study implies that MPs, in synergy with organic pollutants, can be more harmful for marine organisms.

## 1. Introduction

Improper use and disposal of plastics has made plastic pollution a major global environmental concern [1,2,3]. Large quantities of mismanaged plastic wastes enter the oceans worldwide every year [2]. The degradation of these wastes produces plastic fragments or particles less than 5 mm, known as primary microplastics (MPs) [4]. In addition, plastics deliberately manufactured in this size for use in cosmetics or as abrasives are another important source of MPs in the marine environment [5,6]. The ubiquitous distribution of these MPs presents a major threat to various marine organisms and has evolved into an overwhelming challenge for ocean health.

Marine bivalves have been considered particularly susceptible to microplastic pollution, as their strong filter-feeding activity make them readily accumulate MPs from the surrounding seawater [7,8,9]. Under sufficiently high concentrations, MPs result in altered feeding activities, slower growth, and impaired development in bivalves [7]. At the tissue and organ level, gills and the digestive glands are the most important target organs of MPs, in which histopathological alterations and inflammatory response can be observed [10,11,12]. At the cellular level, MPs can trigger a series of stress responses, such as oxidative injury, activation of antioxidant defenses, and destabilization of the lysosomal membrane [10,13]. Overall, these findings clearly indicate that MPs can produce a number of sublethal effects that could reduce the fitness of bivalves. This could impact survival of the individuals, recruitment of new members, and the sustainability of the bivalve population.

It should be noted that marine environments contain a great diversity of pollutants. MPs interact with co-existing pollutants via sorption and desorption. Because of their small size and large surface area, MPs can act as carriers for various pollutants, such as heavy metals, pharmaceuticals, and persistent organic pollutants [6,14,15]. Bivalves are believed to face simultaneous exposure to these chemicals and MPs [16]. While the toxic effects of MPs from single exposure are comparatively well known, the mechanisms and environmental relevance of combined toxicity resulting from the combined pollution deserve further study. Laboratory evidence has shown that MPs play a “carrier” role in increasing the bioaccumulation of sorbed organic pollutants [17,18,19]. Several studies have indicated that MPs exacerbate the adverse effects of organic pollutants [14,15,20,21]. Aquatic organisms suffer from greater histopathological damage or stresses when exposed to organic pollutants (PCBs, PAHs, brominated flame retardants, perfluorinated compounds, or methylmercury) together with MPs than from single pollutants alone [10,22]. For example, co-exposure to bisphenol A (BPA) and polystyrene (PS) nanoparticles generates greater neurotoxic effects in both the central nervous system and the dopaminergic system of zebrafish than to BPA alone [23]. Combined exposure of MPs with organic contaminants can also produce negative effects at genetic levels. Avio et al. [24] observed MP-associated organic contaminants caused irreversible DNA damage in the mussel *Mytilus galloprovincialis*. These studies have clearly indicated the synergistic or additive effects of MPs and organic pollutants. However, some studies contradictorily found that the presence of MPs decrease the toxicity of organic pollutants [25,26]. These inconsistent findings imply the complexity of interactions between MPs and organic pollutants and point to the need for further investigations of their combined toxicity. Currently, few studies have specifically studied the effect of combined exposure of MPs and endocrine disruptors on the gill function in terms of oxidative stress and histopathological changes in this important tissue.

To clarify the effects of exposure to MPs and/or organic pollutants on bivalves, accumulation of reactive oxygen species (ROS) was analyzed in the gills, the first organ. A set of biomarkers, including superoxide dismutase (SOD), peroxidase (POD), catalase (CAT), and peroxidase (POD), involved in oxidative injury and cellular antioxidant defense were studied to reveal the mechanisms counteracting the toxicity in the bivalve. Histopathological damage was observed via microscopic analysis after staining by hematoxylin and eosin. Polystyrene MPs were selected as model contaminants because they are one of the most common MPs in estuaries and coastal areas. Bis(2-ethylhexyl) phthalate (DEHP), one of the most widely used phthalic acid esters (PAEs), was chosen for the representative of endocrine disruptors in order to investigate the synergistic effects with MPs.

## 2. Materials and Methods

### 2.1. Preparations of Organisms and Materials

Healthy *R. philippinarum* (manila clam) and *T. granosa* (blood clam), with a mean shell length of 30.5 ± 1.5 mm and 28.5 ± 1.5 mm, were obtained from Shenzhen (Guangdong, China) and fed *Chlorella vulgaris* in the culture device (10 individuals in 10 L artificial seawater) for 48 h. Before the experiment, 80 *R. philippinarum* and *T. granosa* were acclimatized for one week in plexiglass containers (previously soaked with 10% HNO_3_) with continued aerated and filtered artificial seawater (temperature, 20.5 ± 0.5 °C; pH, 8.1 ± 0.02; salinity, 30.5 ± 1.0) and fed *Chlorella vulgaris* (5.0 × 10^4^ cell mL^−1^) daily in the laboratory [8]. During the acclimatization and culture period, the properties of the artificial seawater were relatively constant. The seawater was changed daily to maintain quality, and no mortality was recorded during the acclimation and exposure period.

**Figure 1:**
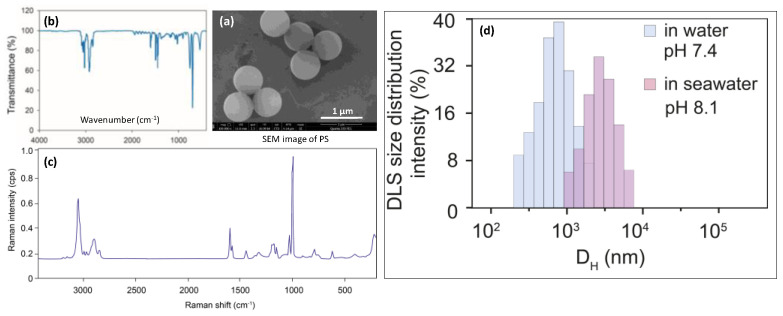
Characterization of PS particles used in this study. SEM image (**a**), infrared spectra (**b**), and Raman spectra (**c**), size distribution of the PS MPs measured by DLS (**d**). Aromatic C-H stretching vibration generated absorption peaks at the wave numbers of 3060 and 3026, and aromatic C=C stretching vibration generated three absorption peaks around the wave numbers of 1600, 1492, and 1452. The Raman spectrum of PS had a typical intense band at 1002 cm^−1^, which was linked to the breathing mode of the aromatic carbon ring.

Polystyrene microplastics beads (approximately 1 μm in diameter, Figure 1, in the concentration of 3.0 mg L^−1^) were used as typical MPs for this study, as PS is one of the most widely applied plastics worldwide and is a commonly encountered MP in marine environments [27,28]. According to previous studies, MPs in the shape of a bead are likely be easier to be ingest and translocate within invertebrates, leading to adverse biological impacts, hence PS microbeads were employed here [29,30]. In addition, the concentration of PS used in this work was considered as representative of the MPs polluted hotspot along coastal environments [31,32]. PS microplastics were synthesized with emulsion polymerization with styrene as a monomer, based on the methods described in Sun et al. [33] and Feng et al. [34]. Briefly, sodium dodecyl sulfate and ammonium persulfate were used as an emulsifier and an initiator, respectively. The synthesized product was transferred to a dialysis bag for removal of redundant styrene monomer, emulsifier, and initiator, and then freeze-dried before use.

The surface morphologies and diameters of the MPs were observed and measured by field emission scanning electron microscopy (FEI-SEM, Quanta 250, FEI Company, Eindhoven, The Netherlands). The FTIR spectra were detected in the 4000–400 cm^−1^ region with a resolution of 4 cm^−1^ by a Nicolet iS10 (Thermo Fisher Scientific Inc., Waltham, WA, USA). The Raman microscope was set up as follows: number of sample scans 3, exposure times 15, background exposure times 512, laser 532 nm, laser energy 8 mW. Each suspected MP was manually located with 10× lens of DXR2 Raman. The average diameter of the PS was 735 ± 87 nm (n = 20). This diameter fell into the size category of nanoplastics (1–1000 nm, Hartmann et al., 2019) and the size range of MPs commonly observed in the coastal environment [35]. The SEM image, infrared spectra, and Raman spectra are shown in Figure 1. Dynamic light scattering (DLS, (Zetasizer, Malvern, UK)) analysis indicated that, in pure water (pH = 7,4), polystyrene microplastics were dispersed homogeneously, with a DLS size of 0.9 μm. In seawater (pH = 8.1), polystyrene MPs were gradually aggregated into approximately 1 to 7.8 μm, with average clusters of 4.9 μm.

### 2.2. Short-Term Exposure of Bivalves to MPs and DEHP

After one week of acclimation, forty healthy *R. philippinarum* and *T. granosa* were randomly assigned to four treatments (in triplicate for 48 h) respectively: Control (with only *R. philippinarum* or *T. granosa*); PS (with 3 mg L^−1^ PS); DEHP (with 50 μg L^−1^ DEHP); and PS + DEHP (with 3 mg L^−1^ PS and 50 μg L^−1^ DEHP). During exposure treatment, ten individuals were randomly translocated into a 12 L plexiglass container (containing 10 L artificial seawater). The concentration of 3 mg L^−1^ PS was approximately 2.6 × 10^12^ particles mL^−1^, respectively. The cultivation conditions were kept the same as the acclimation period, and PS or DEHP were added after feeding *Chlorella vulgaris*.

### 2.3. Measurements of Filtration Rates

The filtration rates assessment was conducted on the basis of previous studies [7,36]. Approximately 150 mL of *Chlorella vulgaris* was added into each plexiglass container, providing a cell density of 5.0 × 10^4^ cell mL^−1^ in artificial seawater. According to the function of microalgae quantity and time, the filtration rate can be calculated. Three repeated samples were collected from each container, in brief, 20 mL seawater was taken before feeding and 1 h after feeding. The concentrations of microalgae were determined by a flow cytometry system (VS-IV, Fluid Imaging Technology, Scarborough, ME, USA). The filtration rates were calculated as follows:

$$\mathrm{Filtration rate}=V/(n\cdot t)\;\times \;\log\;(C_{0}/C_{60})$$

where n is the number of individuals in each container, t is the consumption time (1 h), and C_0_ and C_60_ is the number of microalgae at t = 0 and t = 60, respectively. The results were given as L clam^−1^ h^−1^.

### 2.4. Analysis of SOD, CAT, POD, and MDA in the Gills

The visceral mass of the clams was dissected under low temperature conditions, with three biological samples for each replicate. The collected samples were homogenized in cold phosphate-buffered saline (three organisms per replicate). All pretreated samples were stored at −80 °C until analysis. The enzyme activity, including superoxide dismutase (SOD), catalase (CAT), and peroxidase (POD), were measured using the corresponding commercial assay kit (Nanjing Jiancheng Bioengineering Institute, Nanjing, China). SOD is an antioxidant enzyme, which can not only effectively remove free radicals from the body, but also specifically remove the damage of superoxide free radicals from cells [37]. Generally, the CAT–SOD system is considered to be the first line of defense against the toxicity of reactive oxygen species under adverse stress [38,39]. POD is one of the key enzymes in the enzymatic defense system under adverse conditions, and it cooperates with SOD and CAT to remove excess internal free radicals [14]. Malondialdehyde (MDA), a commonly used index of membrane lipid peroxidation, was determined using an MDA assay kit (Jiancheng, Nanjing, China).

### 2.5. Examining the Accumulation of Reactive Oxygen Species (ROS) in the Gills

In order to visualize O^2−^ and H_2_O_2_ in situ, fresh cut clam tissues were stained with 2′,7′-dichlorodihydrofluorescein diacetate acetyl ester (DCFH-DA; Sigma Aldrich, Burlington, MA, USA) and the ROS fluorescent probe dihydroethidium (DHE; Sigma-Aldrich), respectively [40,41](Waters et al., 2021; Yu et al., 2021). The staining operations were performed according to manufacturer protocols. After incubation, transverse sections were imaged using a fluorescence microscope, with the fluorescence intensity calculated by Image-Pro plus 6.0. In brief, firstly, taking images under 200× of visual field randomly from each treated earthworm slice, we ensured that the background light of each image was consistent. Subsequently, we converted green/red fluorescent monochrome photos into black-and-white images, and then selected the same black as the unified standard (positive control). We analyzed each image to obtain the positive cumulative optical density (IOD) and the pixel area (AREA) of samples, then calculated the average optical density value IOD/AREA (mean density) [42](Adler and Parmryd, 2010).

### 2.6. Histological Analysis of the Gills

To evaluate the histopathological alterations after stressor exposure, the gills of *R. philippinarum* and *T. granosa* were separated and fixed overnight in 10% (*v*/*v*) formaldehyde. Subsequently, each of the gill samples were cut into 3 μm thick sections and embedded in paraffin. The slices were then stained with hematoxylin and eosin (HE) before examination with a microscope, according to Sarasquete and Gutiérrez [43]. The histological changes were assessed using a microscope (3D HISTECH, Pannoramic MIDI, 3D-Histech Ltd., Budapest, Hungary) and the images were analyzed by CaseViewer.

### 2.7. Statistical Analysis

The data were managed using IBM SPSS statistics and OriginPro software. Unless stated otherwise, data in the graphs/plots and tables are displayed as the mean ± s.d. One-way analysis of the variance with an LSD post-hoc test was used for multiple comparisons. When *p* values were less than 0.05, the results were deemed statistically significantly different, but exact *p* values are not shown in the graphs/plots and tables.

## 3. Results and Discussion

### 3.1. Effects of MPs/DEHP Exposures on Filtration Rates of Bivalve Mollusks

**Figure 2:**
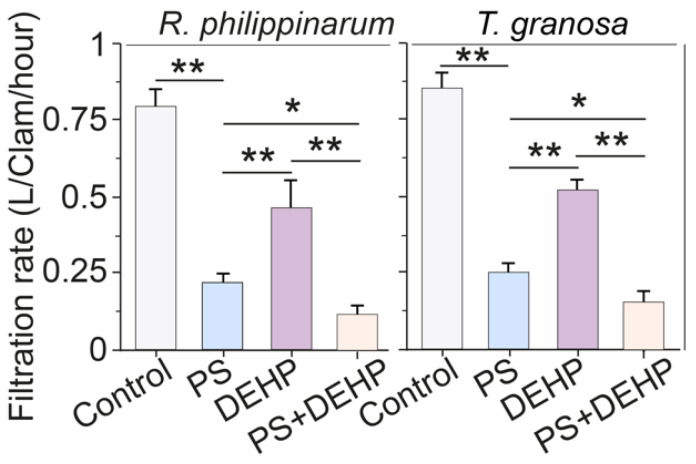
Filtration rates of manila clams (*R. philippinarum*) and blood clams (*T. granosa*) after 48 h of exposure to different PS microplastics and DEHP. Data are presented as mean ± s.d., n = 5. Asterisks above the data indicate significant differences between the two groups, * *p* < 0.05, ** *p* < 0.01.

After a short-term exposure treatment (48 h), the filtration rates (L Clam^−1^ h^−1^) of both types of clams were affected. Exposure of manila and blood clams to the MPs/DEHP tested resulted in significantly higher reductions in the filtration rate (Figure 2), indicating a significant decline in food intake [7,44,45]. As observed in this study, exposure to MPs and DEHP significantly attenuated the filtration rates of both types of clams, and significant differences were observed among the different stressor treatments. These findings are in accordance with the results observed in previous studies [16,46]. 

Specifically, compared to that of control, both types of clams treated with MPs + DEHP had significantly lower filtration rates, which were significantly decreased by approximately 80.3% and 80.4% for manila and blood clams, respectively. Similarly, the filtration rates of manila clams exposed to single MPs (3 mg L^−1^) or DEHP (50 μg L^−1^) declined 70.2% and 38.6%, and for blood clams declined to 77.7% and 41.6% of that of the control, respectively. In this work, for manila clams, the filtration rate in MPs + DEHP group dropped to approximately 34% and 67.9% than that in the single MPs and DEHP group, respectively. For blood clams, compared with that of single treatment, the filtration rate in MPs + DEHP group declined to 12.2% and 66.4%, respectively.

It has been demonstrated that MPs may release endocrine-disrupting compounds to ambient environments such as DEHP, which was found to induce adverse effects in various organisms [47,48,49]. There were significantly lower filtration rates in clams treated with MPs + DEHP, suggesting that the negative impacts induced by single MPs or DEHP might be augmented by the synergic effects of MPs and DEHP [21,50]. Generally, the decrease in the filtration rate after exposure is largely probably owing to the tendency of the tested clams to shut off their valves to avoid continuous exposure [13,51]. As shown in previous studies, *Corbicula fluminea* and *Mytilus edulis L*. closed their valves after exposure to MPs and mercury/bisphenol A, which is an adaptation for bivalves [43,52].

### 3.2. Effects of MPs/DEHP Exposures on Enzymatic and Non-Enzymatic Antioxidant Defenses

In the context of MPs and organic pollutants, exposure is the factor that may induce various responses on the antioxidant markers of aquatic organisms [10,20]. In this study, antioxidant parameters, i.e., SOD, CAT, and POD, in the gill tissues were assessed, due to their critical roles in the uptake and elimination of pollutants, and in different physiological process [9,53]. Lipid peroxidation (MDA content), as the process of ROS (reactive oxygen species) oxidation of biofilm after enhanced oxygen stress [14], was separately evaluated in the gill tissues.

**Figure 3:**
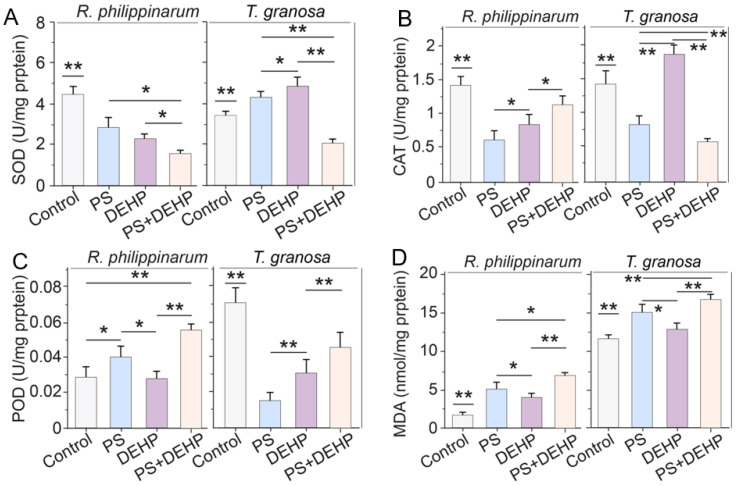
Effects of MPs/DEHP on the activity of antioxidant enzymes (**A**): SOD, superoxide dismutase; (**B**): CAT, catalase; (**C**): POD, peroxidase and lipid peroxidation level (**D**): indicated by malondialdehyde content in manila clams (*R. philippinarum*) and blood clams (*T. granosa*) after 48 h of exposure to different PS microplastics and DEHP. Data are presented as mean ± s.d., n = 5. Asterisks above data indicate significant differences between the two groups, * *p* < 0.05, ** *p* < 0.01.

As an important component of the enzyme antioxidant system, SOD plays an irreplaceable role in the reaction process of homeostatic oxidative stress. The SOD results are shown in Figure 3A. The lowest values were detected in clams exposed to MPs (3 mg L^−1^) + DEHP (50 μg L^−1^), with 1.5 and 2 U mg^−1^ protein in manila and blood clams, respectively, implying that co-exposure to MPs and organic pollutants can impede the free radicals capability of bivalves [54]. Nevertheless, the change trend of SOD activity induced by pollutants exposure was inconsistent in manila and blood clams. For manila clams, both the MPs/DEHP group and the combined exposure group significantly decreased SOD activity. The SOD activity in the MPs + DEHP group was 45.5% and 33.5%, lower than that of MPs (2.8 U mg^−1^ protein) and DEHP (2.3 U mg^−1^ protein) alone. For blood clams, the SOD activity was significantly increased after single exposure, while significantly decreased in MPs + DEHP treatment, which was 40% lower than that of the control (3.4 U mg^−1^ protein).

The results from CAT are shown in Figure 3B. The exposure of clams to MPs or MPs + DEHP decreased the CAT antioxidant activity after 48 h compared with control. The reduction in CAT activity suggests that MPs alone or combined with organic pollutants might cause actual inhibitory effects on the CAT–SOD system after a short-term exposure [14], whereas increased activity was observed when blood clams were exposed to DEHP alone. For manila clams, exposure to MPs/DEHP alone or combined significantly decreased CAT activity, and co-exposure to MPs + DEHP led to less impact on CAT activity (1.1 U mg^−1^ protein), which was 89.6% and 36.9% higher than that of MPs (0.6 U mg^−1^ protein) and DEHP (0.8 U mg^−1^ protein) alone. For blood clams, MPs and MPs + DEHP exposure significantly decreased CAT activity, however, CAT activity was significantly promoted in the DEHP group (1.9 U mg^−1^ protein), which was 30% higher than that of the control (1.4 U mg^−1^ protein).

Total POD activities varied from 0.028 to 0.06 U mg^−1^ protein in manila clams and from 0.012 to 0.072 U mg^−1^ protein in blood clams (Figure 3C), with the lowest value being recorded in the DEHP and MPs group, respectively, after 48 h of exposure. For manila clams, MPs (0.04 U mg^−1^ protein) or MPs + DEHP (0.055 U mg^−1^ protein) treatment significantly increased POD activity, while DEHP exposure alone (0.029 U mg^−1^ protein) had no effect. For blood clams, POD activities were largely decreased by all treatments; additionally, the decrease of POD activity (0.055 U mg^−1^ protein) in the MPs + DEHP group was lower than that of the MPs (0.045 U mg^−1^ protein) or DEHP (0.031 U mg^−1^ protein) group. 

The lipid peroxidation (indicated by MDA content) results display a trend to increase throughout the exposure tests for both types of clams (manila and blood clams) (Figure 3D). The lowest values were measured in control organisms (1.51 and 11.5 nM mg^−1^ protein, respectively) and the highest values (6.8 and 16.6 nM mg^−1^ protein, respectively) were observed in organisms exposed to MPs + DEHP after 48 h of exposure. For manila clams, the MDA content in clams exposed to MPs + DEHP was 36.1% and 79.9% higher than those exposed to MPs and DEHP alone, respectively. For blood clams, the content of MDA in the MPs + DEHP group was 11.1% and 28.9% higher than that of single group, respectively. 

When bivalves are exposed to MPs or organic pollutants, ROS, i.e., O^2−^, H_2_O_2_, or HO, will be over-accumulated, which can directly cause LPO damage [15,55]. The excessive ROS thus leads to an increase of MDA, which is one of the byproducts of LPO [15]. Generally, the present findings of growth inhibition, antioxidant enzyme activities (SOD, CAT and POD) alteration, and MDA increase in single/combined toxicity indicate that the presence of microplastics and organic pollutants could cause the gills of bivalves to accelerate the production of ROS [12].

### 3.3. Effects of MPs/DEHP Exposures on ROS Accumulation

**Figure 4:**
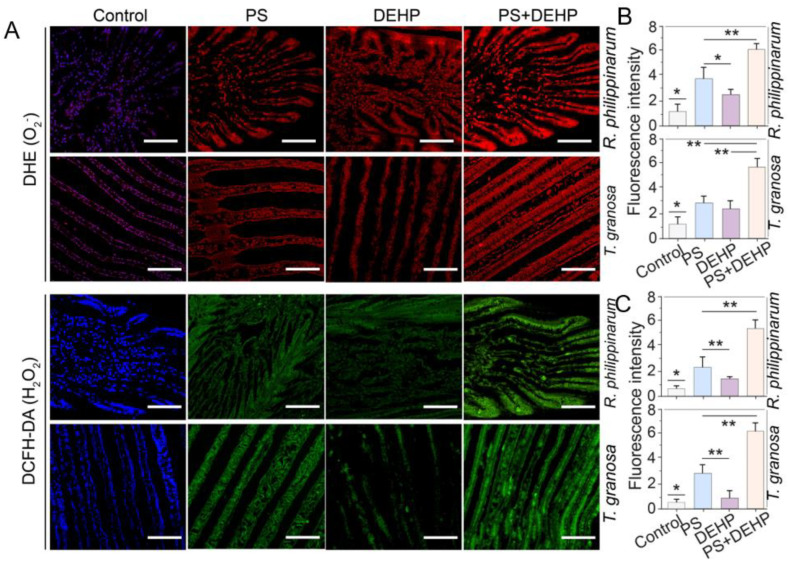
Effects of MPs/DEHP on the accumulation of ROS in gills of manila clams (*R. philippinarum*) and blood clams (*T. granosa*) after 48 h exposure to different PS microplastics and DEHP (**A**). Red and green fluorescence indicate O^2−^ and H_2_O_2_, respectively, representative pictures. Data are presented as mean ± s.d, n = 5. The fluorescence intensity of red (**B**) and green (**C**) values were calculated from six different pictures for each data, n = 6, scale bar 200 μm. Asterisks above data indicate significant differences between the two groups, * *p* < 0.05, ** *p* < 0.01.

To assess the accumulations of ROS in gills of manila clams (*R. philippinarum*) and blood clams (*T. granosa*), O^2−^ and H_2_O_2_ were detected using DHE and DCFH-DA. The ROS (O^2−^ and H_2_O_2_) staining observations revealed that the ROS concentration in gills of manila and blood clams were significantly enhanced by MPs/DEHP exposure for 48 h (Figure 4). This is consistent with the results of MDA increase, and is also in accordance with previous studies’ findings [54,56]. For manila clams, regardless of whether MPs and DEHP exposure was alone or combined, ROS accumulation occurred in the gill tissues. The combined exposure showed higher accumulation levels, followed by MPs alone exposure. Compared to the control, the O^2−^ and H_2_O_2_ concentrations in MPs + DEHP were approximately 6 and 8.6 times higher, respectively (Figure 4B,C). For blood clams, similar results were observed, in which MPs and DEHP exposed alone or combined, obvious ROS accumulation was detected in the gill tissues. Co-exposure led to higher accumulation levels, followed by MPs alone exposure. Compared to the control, the O^2−^ and H_2_O_2_ concentrations in MPs + DEHP were approximately 5 and 12.2 times higher, respectively (Figure 3B,C).

In general, MPs exposure in organisms can disrupt the redox homeostasis and cause oxidative challenges by rapidly accumulating ROS concentrations in tissue [38]. In this work, ingestion of microplastics by bivalves induced oxidative stress that destabilized the homeostasis and produced free radical and leads to increases ROS in gill tissue of all treated clams. Polystyrene microplastics have been found to increase the ROS production level in various aquatic organisms [56,57,58]. This could possibly be due to the insufficient elimination of free radicals in clams, eventually causing oxidative damage and histopathological changes [35,58]. Over-accumulated ROS affect the antioxidant system response and cause oxidative stress [59]. As a result, short-term dietary exposure to polypropylene microplastics and/or organic pollutant (DEHP) influences the antioxidant defense system of both manila and blood clams.

### 3.4. Effects of MPs/DEHP Exposures on Histological Alterations

**Figure 5:**
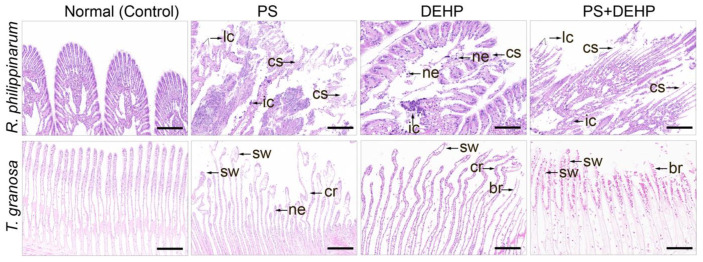
Representative images of haemotoxylin/eosin-stained sections of gill extracted from manila clams (*R. philippinarum*) and blood clams (*T. granosa*). Normal structure shown in the control group, histopathological changes occurred after single/co-exposure to polystyrene MPs/DEHP, Scale bar 200 μm. cs: ciliary structure destruction; lc: loss of contact between gill filaments; ic: inflammatory infiltration; ne: necrosis; cr: curly. sw: swollen; lh: rare in hemocytes; br: broken; ne: necrosis.

Obvious histopathological changes were observed in the gills of both types of clams, as a result of 48 h actual toxicity tests, after the microscopic examination of the gill tissues sections (Figure 5). Co-exposure to MPs and DEHP in particular caused discrete pathologies in the gills. No histopathological alterations were found in the gill tissues of the control groups, which had normal structure, displaying basic features of the primary and secondary lamellae with typical pillar, chloride cells in the epithelium, and hemocytes throughout the basal to frontal zones (especially in blood clams). MPs exposure has been reported to cause remarkable tissue pathologies in various aquatic organisms, i.e., *Crassostrea gigas*, *Mytilus* spp., *Pomacea paludosa*, and *Mytilus edulis*. [11,12,60]. Similar effects were also observed to be induced by combined MPs and DEHP. For example, polystyrene plastics particles and DEHP co-exposure caused significant histological damages in *Micropterus salmoides* [61].

However, in comparison to single treatment of DEHP alone, the histopathological degree was more serious in the MPs group and the gill filaments were morphologically changed. For manila clams, pathological changes occurred in the tissues, such as ciliary structure destruction (cs), loss of contact between gill filaments (lc), inflammatory infiltration (ic), and necrosis (ne). For blood clams, there were curly (cr) and swollen (sw) gill filaments in the frontal lobe, and rare in hemocytes (lh). Co-exposure to MPs + DEHP led to abnormal gill structure, mainly manifested in the gill filament terminal being swollen (sw) or broken (br), and cell necrosis (ne). In addition, compared with single exposure to MPs/DEHP, more obvious morphological changes were found in the gills of the MPs + DEHP group, which completely lost their original features and became necrotic. 

It has been demonstrated that MPs and DEHP exposure can interfere with carbohydrate metabolism and ROS elimination of tested organisms [62,63] (Brate et al., 2018; Romano et al., 2018). This leads to significant pathologies alterations, such as inflammation and cell necrosis, in both vertebrate and invertebrate species following MPs or organic pollutants exposure [7,61] (Sikdokur et al., 2020; Liao et al., 2022). The histopathological alterations that may occur in tested organisms can vary depending on biotic and abiotic factors, e.g., animal (species, life stage, tolerance capability etc.), pollutant (type and characteristics), and exposure condition (concentration, duration, and pathway) [3,64,65,66]. In this study, the pathological changes that occurred in the gills of both types of clams may be attributed as a cause of attenuated filtration rate.

## 4. Conclusions

The results of the current study indicate that both *R. philippinarum* and *T. granosa* are vulnerable to short-term microplastics and DEHP exposure. The filtration rate of *R. philippinarum* and *T. granosa* were significantly decreased as a result of the exposure to MPs and DEHP, especially co-exposure treatment, suggesting single/combined exposure causes significant feeding inhibition. In regard to antioxidant enzymes activities, like SOD, CAT, and POD, the gills of both types of clams were considerably changed as a result of the MPs and DEHP exposure. The lipid peroxidation level (MDA) occurred in single/combined toxicity, implying that the presence of MPs and DEHP could induce the gill of bivalves to accelerate the production of ROS. Further analysis confirmed the over-accumulation of ROS (O^2−^ and H_2_O_2_) in gill tissues of *R. philippinarum* and *T. granosa*, thus leading to oxidative damage and histopathological changes, which results in a sharp decrease of the filtration rate. 

Considering the widespread pollution of marine ecosystems by MPs and organic pollutants, once in severely contaminated environments, bivalves are inevitably at risk of suffering adverse effects deriving from both MPs and organic pollutants. However, it should be noted that the size of MPs was around 700 nm in this study, which is larger than the typical nanoplastics (1 to 100 nm) [67]. The scope of this study is limited, as it only investigated one size of nanoplastic. Size is a critical parameter that determines the toxicity of nanoplastics [68,69]. To fully understand the ecological impacts of nanoplastics and the associated organic pollutants, it is imperative that more research is conducted to investigate the effect of size. Greater toxicity is expected for smaller particles because of their high specific surface area and their ability to cross cell membranes where they may cause oxidative stress within the cell.

## Data Availability

Not applicable.

## References

[1] Schmaltz E., Melvin E.C., Diana Z., Gunady E.F., Rittschof D., Somarelli J.A., Virdin J., Dunphy-Daly M.M. (2020). Plastic pollution solutions: Emerging technologies to prevent and collect marine plastic pollution. Environ. Int..

[2] MacLeo M., Arp H.P.H., Tekman M.B., Jahnke A. (2021). The global threat from plastic pollution. Science.

[3] Wang C., Zhao J., Xing B. (2021). Environmental source, fate, and toxicity of microplastics. J. Hazard. Mater..

[4] Law K.L., Thompson R.C. (2014). Microplastics in the seas. Science.

[5] Turner A. (2020). Foamed Polystyrene in the Marine Environment: Sources, Additives, Transport, Behavior, and Impacts. Environ. Sci. Technol..

[6] Wang Y., Yang Y., Liu X., Zhao J., Liu R., Xing B. (2021). Interaction of Microplastics with Antibiotics in Aquatic Environment: Distribution, Adsorption, and Toxicity. Environ. Sci. Technol..

[7] Sikdokur E., Belivermis M., Sezer N., Pekmez M., Bulan O.K., Kilic O. (2020). Effects of microplastics and mercury on manila clam Ruditapes philippinarum: Feeding rate, immunomodulation, histopathology and oxidative stress. Environ. Pollut..

[8] Jiang W.W., Fang J.H., Du M.R., Gao Y.P., Fang J.G., Jiang Z.J. (2022). Microplastics influence physiological processes, growth and reproduction in the Manila clam, Ruditapes philippinarum. Environ. Pollut..

[9] Zhou Z., Ni X., Chen S., Wu Z., Tang J., Su Y., Wang X., Wang L. (2022). Ingested microplastics impair the metabolic relationship between the giant clam Tridacna crocea and its symbionts. Aquat. Toxicol..

[10] Paul-Pont I., Lacroix C., Gonzalez Fernandez C., Hegaret H., Lambert C., Le Goic N., Frere L., Cassone A.-L., Sussarellu R., Fabioux C. (2016). Exposure of marine mussels Mytilus spp. to polystyrene microplastics: Toxicity and influence on fluoranthene bioaccumulation. Environ. Pollut..

[11] Teng J., Zhao J., Zhu X., Shan E., Zhang C., Zhang W., Wang Q. (2021). Toxic effects of exposure to microplastics with environmentally relevant shapes and concentrations: Accumulation, energy metabolism and tissue damage in oyster Crassostrea gigas. Environ. Pollut..

[12] Jeyavani J., Sibiya A., Gopi N., Mahboob S., Riaz M.N., Vaseeharan B. (2022). Dietary consumption of polypropylene microplastics alter the biochemical parameters and histological response in freshwater benthic mollusc Pomacea paludosa. Environ. Res..

[13] Monteiro R., Costa S., Coppola F., Freitas R., Vale C., Pereira E. (2019). Toxicity beyond accumulation of Titanium after exposure of Mytilus galloprovincialis to spiked seawater. Environ. Pollut..

[14] Yang W., Gao X., Wu Y., Wan L., Tan L., Yuan S., Ding H., Zhang W. (2020). The combined toxicity influence of microplastics and nonylphenol on microalgae Chlorella pyrenoidosa. Ecotoxicol. Environ. Saf..

[15] Zhang J., Meng H., Kong X., Cheng X., Ma T., He H., Du W., Yang S., Li S., Zhang L. (2021). Combined effects of polyethylene and organic contaminant on zebrafish (Danio rerio): Accumulation of 9-Nitroanthracene, biomarkers and intestinal microbiota. Environ. Pollut..

[16] Guo X., Cai Y., Ma C., Han L., Yang Z. (2021). Combined toxicity of micro/nano scale polystyrene plastics and ciprofloxacin to Corbicula fluminea in freshwater sediments. Sci. Total Environ..

[17] Besseling E., Wegner A., Foekema E.M., Van Den Heuvel-Greve M.J., Koelmans A.A. (2013). Effects of microplastic on fitness and PCB bioaccumulation by the lugworm Arenicola marina (L.). Environ. Sci. Technol..

[18] Wardrop P., Shimeta J., Nugegoda D., Morrison P.D., Miranda A., Tang M., Clarke B.O. (2016). Chemical pollutants sorbed to ingested microbeads from personal care products accumulate in fish. Environ. Sci. Technol..

[19] Zhao H.J., Xu J.K., Yan Z.H., Ren H.Q., Zhang Y. (2020). Microplastics enhance the developmental toxicity of synthetic phenolic antioxidants by disturbing the thyroid function and metabolism in developing zebrafish. Environ. Int..

[20] O’Donovan S., Mestre N.C., Abel S., Fonseca T.G., Carteny C.C., Cormier B., Keiter S.H., Bebianno M.J. (2018). Ecotoxicological Effects of Chemical Contaminants Adsorbed to Microplastics in the Clam Scrobicularia plana. Front. Mar. Sci..

[21] Shi Q., Tang J., Wang L., Liu R., Giesy J.P. (2021). Combined cytotoxicity of polystyrene nanoplastics and phthalate esters on human lung epithelial A549 cells and its mechanism. Ecotoxicol. Environ. Saf..

[22] Rainieri S., Conlledo N., Larsen B.K., Granby K., Barranco A. (2018). Combined effects of microplastics and chemical contaminants on the organ toxicity of zebrafish (Danio rerio). Environ. Res..

[23] Chen Q., Yin D., Jia Y., Schiwy S., Legradi J., Yang S., Hollert H. (2017). Enhanced uptake of BPA in the presence of nanoplastics can lead to neurotoxic effects in adult zebrafish. Sci. Total Environ..

[24] Avio C.G., Gorbi S., Milan M., Benedetti M., Fattorini D., d’Errico G., Regoli F. (2015). Pollutants bioavailability and toxicological risk from microplastics to marine mussels. Environ. Pollut..

[25] Oliveira M., Ribeiro A., Hylland K., Guilhermino L. (2013). Single and combined effects of microplastics and pyrene on juveniles (0+ group) of the common goby Pomatoschistus microps (Teleostei, Gobiidae). Ecol. Indic..

[26] Rehse S., Kloas W., Zarfl C. (2018). Microplastics reduce short-term effects of environmental contaminants. Part I: Effects of bisphenol A on freshwater zooplankton are lower in presence of polyamide particles. Int. J. Environ. Res. Public Health.

[27] Courtene-Jones W., Quinn B., Ewins C., Gary S.F., Narayanaswamy B.E. (2019). Consistent microplastic ingestion by deep-sea invertebrates over the last four decades (1976–2015), a study from the North East Atlantic. Environ. Pollut..

[28] Kutralam-Muniasamy G., Perez-Guevara F., Elizalde-Martinez I., Shruti V.C. (2020). Review of current trends, advances and analytical challenges for microplastics contamination in Latin America. Environ. Pollut..

[29] Hanachi P., Karbalaei S., Yu S. (2021). Combined polystyrene microplastics and chlorpyrifos decrease levels of nutritional parameters in muscle of rainbow trout (Oncorhynchus mykiss). Environ. Sci. Pollut. Res..

[30] Huang W., Wang X., Chen D., Xu E.G., Luo X., Zeng J., Huan T., Li L., Wang Y. (2021). Toxicity mechanisms of polystyrene microplastics in marine mussels revealed by high-coverage quantitative metabolomics using chemical isotope labeling liquid chromatography mass spectrometry. J. Hazard. Mater..

[31] Goldstein M.C., Rosenberg M., Cheng L. (2012). Increased oceanic microplastic debris enhances oviposition in an endemic pelagic insect. Biol. Lett..

[32] Ivar do Sul J.A., Costa M.F., Fillmann G. (2014). Microplastics in the pelagic environment around oceanic islands of the Western Tropical Atlantic Ocean. Water Air Soil Pollut..

[33] Sun X.D., Yuan X.Z., Jia Y., Feng L.J., Zhu F.P., Dong S., Liu J.J., Kong X.P., Tian H.Y., Duan J.L. (2020). Differentially charged nanoplastics demonstrate distinct accumulation in Arabidopsis thaliana. Nat. Nanotechnol..

[34] Feng L.J., Wang J.J., Liu S.C., Sun X.D., Yuan X.Z., Wang S.G. (2018). Role of extracellular polymeric substances in the acute inhibition of activated sludge by polystyrene nanoparticles. Environ. Pollut..

[35] Chai B., Li Y., Wang L., Zhang X.T., Wan Y.P., Chen F., Pan K. (2022). Microplastic contamination on the beaches of South China. Front. Mar. Sci..

[36] Riisgard H.U., Pleissner D., Lundgreen K., Larsen P.S. (2013). Growth of mussels Mytilus edulis at algal (Rhodomonas salina) concentrations below and above saturation levels for reduced filtration rate. Mar. Biol. Res..

[37] Vale G., Mehennaoui K., Cambier S., Libralato G., Jomini S., Domingos R.F. (2016). Manufactured nanoparticles in the aquatic environment-biochemical responses on freshwater organisms: A critical overview. Aquat. Toxicol..

[38] Winston G.W., Digiulio R.T. (1991). Prooxidant and antioxidant mechanisms in aquatic organisms. Aquat. Toxicol..

[39] Britto R.S., Nascimento J.P., Serodre T., Santos A.P., Soares A.M.V.M., Furtado C., Ventura-Lima J., Monserrat J.M., Freitas R. (2021). Oxidative stress in Ruditapes philippinarum after exposure to different graphene oxide concentrations in the presence and absence of sediment. Comp. Biochem. Physiol. C-Toxicol. Pharmacol..

[40] Waters E.C.T., Baark F., Yu Z., Mota F., Eykyn T.R., Yan R., Southworth R. (2022). Detecting Validated Intracellular ROS Generation with F-18-dihydroethidine-Based PET. Mol. Imaging Biol..

[41] Yu D., Zha Y., Zhong Z., Ruan Y., Li Z., Sun L., Hou S. (2021). Improved detection of reactive oxygen species by DCFH-DA: New insight into self-amplification of fluorescence signal by light irradiation. Sens. Actuators B-Chem..

[42] Adler J., Parmryd I. (2010). Quantifying colocalization by correlation: The pearson correlation coefficient is cuperior to the mander’s overlap coefficient. Cytom. Part A.

[43] Sarasquete C., Gutierrez M. (2005). New tetrachromic VOF stain (Type III-G.S) for normal and pathological fish tissues. Eur. J. Histochem..

[44] Esperanza M., Seoane M., Servia M.J., Cid A. (2020). Effects of Bisphenol A on the microalga Chlamydomonas reinhardtii and the clam Corbicula fluminea. Ecotoxicol. Environ. Saf..

[45] Zha S., Tang Y., Shi W., Liu H., Sun C., Bao Y., Liu G. (2022). Impacts of four commonly used nanoparticles on the metabolism of a marine bivalve species, Tegillarca granosa. Chemosphere.

[46] Oliveira P., Antao Barboza L.G., Branco V., Figueiredo N., Carvalho C., Guilhermino L. (2018). Effects of microplastics and mercury in the freshwater bivalve Corbicula fluminea (Muller, 1774): Filtration rate, biochemical biomarkers and mercury bioconcentration. Ecotoxicol. Environ. Saf..

[47] Coffin S., Lee I., Gan J., Schlenk D. (2019). Simulated digestion of polystyrene foam enhances desorption of diethylhexyl phthalate (DEHP) and In vitro estrogenic activity in a size-dependent manner. Environ. Pollut..

[48] Molino C., Filippi S., Stoppiello G.A., Meschini R., Angeletti D. (2019). In Vitro evaluation of cytotoxic and genotoxic effects of Di(2-ethylhexyl)-phthalate (DEHP) on European sea bass (Dicentrarchus labrax) embryonic cell line. Toxicol. Vitr..

[49] Rios-Fuster B., Alomar C., Capo X., Gonzalez G.P., Martinez R.M.G., Rojas D.L.S., Silva M., Hernando P.F., Sole M., Freitas R. (2022). Assessment of the impact of aquaculture facilities on transplanted mussels (Mytilus galloprovincialis): Integrating plasticizers and physiological analyses as a biomonitoring strategy. J. Hazard. Mater..

[50] Heinder F.M., Alajmi F., Huerlimann R., Zeng C., Newman S.J., Vamvounis G., van Herwerden L. (2017). Toxic effects of polyethylene terephthalate microparticles and Di(2-ethylhexyl)phthalate on the calanoid copepod, Parvocalanus crassirostris. Ecotoxicol. Environ. Saf..

[51] Araujo C.V.M., Gomez L., Silva D.C.V.R., Pintado-Herrera M.G., Lara-Martin P.A., Hampel M., Blasco J. (2019). Risk of triclosan based on avoidance by the shrimp Palaemon varians in a heterogeneous contamination scenario: How sensitive is this approach?. Chemosphere.

[52] Wegner A., Besseling E., Foekema E.M., Kamermans P., Koelmans A.A. (2012). Effects of nanopolystyrene on the feeding behavior of the blue mussel (*Mytilus edulis* L.). Environ. Toxicol. Chem..

[53] Ozdilek S.Y., Demir N., Gurkan S.E. (2019). Assessment of pollution biomarker and stable isotope data in Mytilus galloprovincialis tissues. Environ. Monit. Assess..

[54] Guilhermino L., Vieira L.R., Ribeiro D., Tavares A.S., Cardoso V., Alves A., Almeida J.M. (2018). Uptake and effects of the antimicrobial florfenicol, microplastics and their mixtures on freshwater exotic invasive bivalve Corbicula fluminea. Sci. Total Environ..

[55] Piddington D.L., Fang F.C., Laessig T., Cooper A.M., Orme I.M., Buchmeier N.A. (2001). Cu,Zn superoxide dismutase of Mycobacterium tuberculosis contributes to survival in activated macrophages that are generating an oxidative burst. Infect. Immun..

[56] Han Y., Zhou W., Tang Y., Shi W., Shao Y., Ren P., Zhang J., Xiao G., Sun H., Liu G. (2021). Microplastics aggravate the bioaccumulation of three veterinary antibiotics in the thick shell mussel Mytilus coruscus and induce synergistic immunotoxic effects. Sci. Total Environ..

[57] Mao Y., Ai H., Chen Y., Zhang Z., Zeng P., Kang L., Li W., Gu W., He Q., Li H. (2018). Phytoplankton response to polystyrene microplastics: Perspective from an entire growth period. Chemosphere.

[58] Zhao T., Tan L., Zhu X., Huang W., Wang J. (2020). Size-dependent oxidative stress effect of nano/micro-scaled polystyrene on Karenia mikimotoi. Mar. Pollut. Bull..

[59] Trestrail C., Nugegoda D., Shimeta J. (2020). Invertebrate responses to microplastic ingestion: Reviewing the role of the antioxidant system. Sci. Total Environ..

[60] Revel M., Lagarde F., Perrein-Ettajani H., Bruneau M., Akcha F., Sussarellu R., Rouxel J., Costil K., Decottignies P., Cognie B. (2019). Tissue-Specific Biomarker Responses in the Blue Mussel Mytilus spp. Exposed to a Mixture of Microplastics at Environmentally Relevant Concentrations. Front. Environ. Sci..

[61] Liao H., Liu S., Junaid M., Gao D., Ai W., Chen G., Wang J. (2022). Di-(2-ethylhexyl) phthalate exacerbated the toxicity of polystyrene nanoplastics through histological damage and intestinal microbiota dysbiosis in freshwater Micropterus salmoides. Water Res..

[62] Brate I.L.N., Blazquez M., Brooks S.J., Thomas K.V. (2018). Weathering impacts the uptake of polyethylene microparticles from toothpaste in Mediterranean mussels (*M. galloprovincialis*). Sci. Total Environ..

[63] Romano N., Ashikin M., Teh J.C., Syukri F., Karami A. (2018). Effects of pristine polyvinyl chloride fragments on whole body histology and protease activity in silver barb Barbodes gonionotus fry. Environ. Pollut..

[64] Prata J.C., da Costa J.P., Lopes I., Duarte A.C., Rocha-Santos T. (2020). Environmental exposure to microplastics: An overview on possible human health effects. Sci. Total Environ..

[65] Kim J.-H., Yu Y.-B., Choi J.-H. (2021). Toxic effects on bioaccumulation, hematological parameters, oxidative stress, immune responses and neurotoxicity in fish exposed to microplastics: A review. J. Hazard. Mater..

[66] Rahman A., Sarkar A., Yadav O.P., Achari G., Slobodnik J. (2021). Potential human health risks due to environmental exposure to nano-and microplastics and knowledge gaps: A scoping review. Sci. Total Environ..

[67] Koelmans A.A. (2015). Modeling the role of microplastics in bioaccumulation of organic chemicals to marine aquatic organisms. A critical review. Mar. Anthropog. Litter.

[68] Miao L., Hou J., You G., Liu Z., Liu S., Li T., Qu H. (2019). Acute effects of nanoplastics and microplastics on periphytic biofilms depending on particle size, concentration and surface modification. Environ. Pollut..

[69] Ning Q., Wang D., An J., Ding Q., Huang Z., Zou Y., You J. (2022). Combined effects of nanosized polystyrene and erythromycin on bacterial growth and resistance mutations in Escherichia coli. J. Hazard. Mater..

